# Polyamine Metabolism Promotes the Progression of Thyroid Carcinoma by Regulating the Immune Phenotype of Tumor Associated Macrophages

**DOI:** 10.1002/smmd.70009

**Published:** 2025-05-16

**Authors:** Haoran Ding, Lingling Xue, Shaoshi Zhu, Ziyu Wang, Huan Gui, Guang Zhang, Ning Tang, Haozhen Ren, Dayu Chen

**Affiliations:** ^1^ Division of Thyroid Surgery Department of General Surgery Nanjing Drum Tower Hospital The Affiliated Hospital of Medical School Nanjing University Nanjing China; ^2^ Division of Hepatobiliary and Transplantation Surgery Department of General Surgery Nanjing Drum Tower Hospital Clinical College of Nanjing University of Chinese Medicine Nanjing China; ^3^ College of Medicine University of Illinois Chicago Illinois USA; ^4^ Jiamusi University Jiamusi China; ^5^ Joint Research Unit Physiology of Nutritional Adaptation INRAE (National Research Institute for Agricultural, Food and Environmental) & University of Nantes Nantes France; ^6^ Department of Pharmacy Nanjing Drum Tower Hospital The Affiliated Hospital of Nanjing University Medical School Nanjing China; ^7^ School of Pharmacy Faculty of Medicine Macau University of Science and Technology Macau China

**Keywords:** immunotherapy, macrophage, polyamine metabolism, thyroid carcinoma

## Abstract

Polyamine metabolism is a key regulator of cellular proliferation and immune modulation, and its dysregulation is implicated in multiple carcinoma pathogenesis. Macrophages also greatly influence tumor progression by regulating the immune microenvironment. However, the role of polyamine metabolism in thyroid cancer macrophages remains understudied. This study explores the connection between polyamine metabolism and macrophages in thyroid cancer. Using the THCA dataset gene expression analysis and single‐cell data, we identified macrophage subpopulations. We assessed immune scores, matrix scores, immune checkpoint scores, and immune cell types using Bulk‐RNA data and the TIDE platform to predict immune checkpoint inhibitor responses. Clinical specimens validated our findings. Our results show a significant association between polyamine metabolism and the clinical and biological characteristics of thyroid cancer, including macrophage trajectory. Notably, macrophage subpopulations affected by polyamine metabolism have strong prognostic value, especially in immunotherapy patients. We found that changes in these subpopulations correlate with thyroid cancer development, and tumor tissue can regulate macrophage polyamine metabolism. This study provides new insights into how polyamine metabolism affects macrophages and the tumor microenvironment, influencing tumor growth and anti‐tumor immune responses in thyroid cancer.

## Graphical abstract

This study reveals that polyamine metabolism significantly impacts thyroid carcinoma progression by modulating tumor‐associated macrophage phenotypes, influencing tumor growth and immune responses.

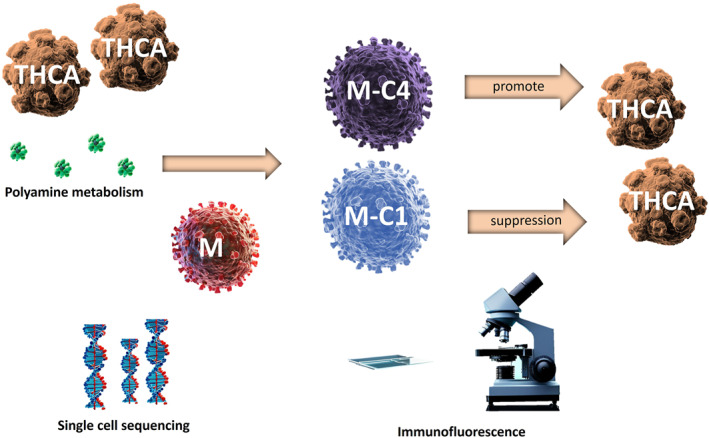

**Summary**

- Polyamine metabolism is linked to THCA progression through its effects on tumor‐associated macrophages.
- Macrophage subpopulations influenced by polyamine metabolism show strong prognostic value and impact immunotherapy response.
- Clinical validation suggests that targeting polyamine metabolism could improve treatment efficacy in advanced THCA.

**Abbreviations**
BRAF: V‐raf Murine Sarcoma Viral Oncogene Homolog B1
CSF‐1: Colony Stimulating Factor‐1
CTLA: Cytotoxic T‐Lymphocyte Antigen 4
FOXP3: Forkhead Box Protein 3
GSVA: Gene Set Variation Analysis
ICB: immune checkpoint block
IFN γ: Interferon γ
IRF7: Interferon Regulator Factor 7
KRAS: Kirsten Ratsarcoma Sarcoma Viral Oncogene Homolog
MYC: Myelocytomatosis Viral Oncogene
NMF: Non‐negative matrix factorization
ODC1: Ornithine Decarboxylase 1
PD1: Programmed Cell Death Protein 1
PSMA2: Proteasome Subunit Alpha Type‐2
PSME2: Proteasome Activator Complex Subunit 2
ROS: Reactive Oxygen Species
SPI1: Spleen Focus Forming Virus Proviral Integration Oncogene 1
STAT1: Signal Transducer and Activator of Transcription 1
TAMs: Tumor‐Associated Macrophages
TCR: T Cell Receptor
TGFB: Transforming Growth Factor Beta
THCA: Thyroid Carcinoma
TLRs: Toll Like Receptors
TME: Tumor Microenvironment
TNF α: Tumor Necrosis Factor α
Treg: Regulatory T Cells

## 1 Introduction

With the continuous advancement of ultrasound technology and increasing awareness of medical issues among the population, worldwide data have shown that the incidence of thyroid carcinoma (THCA) has increased significantly and has become a trend of high incidence of cancer since the early 1970s [1, 2, 3]. THCA is generally divided into four histological types, including well‐differentiated papillary THCA and follicular THCA, which are more common in thyroid cancers and generally have a favorable prognosis; then, there are poorly differentiated THCA and medullary THCA [4, 5]. Surgery is the best treatment for THCA, followed by radiotherapy and chemotherapy [6, 7]. However, when it comes to advanced THCA, standardized treatments often prove ineffective [8]. Despite extensive studies on genetic alterations (e.g., BRAF and RAS mutations) in THCA, the metabolic reprogramming of immune cells within the tumor microenvironment, particularly macrophages, remains poorly characterized. Nevertheless, researchers have developed various treatment approaches based on existing findings, such as tyrosine kinase inhibitors and anti‐angiogenic drugs. Nevertheless, the application of these treatment methods is limited by drug resistance and poor efficacy in some patients [9, 10]. Consequently, the lack of highly effective treatment methods for patients with advanced THCA remains a major obstacle. Therefore, it is vital for researchers to further study the mechanism of thyroid development, promote the discovery of new drugs, and improve the efficacy of existing treatments.

Tumor microenvironment (TME) is a complex tumor ecosystem, which is composed of complex interactions between tumor cells, infiltrating immune cells, and extracellular matrix [11]. Among the various inflammatory cells that constitute the TME, tumor‐associated macrophages (TAMs) are involved in tumor suppression and growth [12]. Studies have confirmed that excessive infiltration of macrophages is closely related to poor prognosis of tumors [13]. Macrophages mainly consist of M1‐type and M2‐type, among which M1‐type macrophages are activated by interferon γ (IFNγ) and Toll‐like receptors (TLRs) after stimulation, and release tumor necrosis factor α (TNF‐α) and other pro‐inflammatory factors, thus producing an immune killing effect. Conversely, M2‐type macrophages execute immune regulatory functions by secreting IL‐4, IL‐13, and IL‐10, thereby promoting tissue regeneration and inhibiting immune damage [14, 15, 16]. Due to signaling regulation in TME, the proportion of M2‐type macrophages is generally much higher than M1‐type macrophages, which are responsible for improving tumor survival, proliferation, angiogenesis, and metastasis, etc [12, 17]. Moreover, TAMs inhibit tumor immune response mediated by other immune cells in TME by releasing immunosuppressive factors [18]. Apart from M1 and M2 types, various other macrophage populations typically regulate biological processes such as tissue development and material metabolism, operating through the CSF‐1 pathway [19]. Therefore, TAMs constitute a heterogeneous group of macrophage subpopulations. Understanding the intricate interactions and mechanisms between tumor cells and macrophages provides novel insights into tumor immunotherapy. Leveraging this knowledge can potentially enhance the prognosis of tumor patients, particularly those with increased macrophage infiltration.

Putrescine, spermidine, and spermine are essential polyamines present in mammalian cells and play critical roles in diverse cellular processes by interacting with negatively charged macromolecules [20, 21, 22]. These processes encompass gene regulation, cell proliferation, differentiation, and immune system modulation [23]. Notably, polyamine metabolism is intricately linked to the sustained proliferation and metastasis of cancer cells. Numerous oncogenes, such as MYC, KRAS, and BRAF, are implicated in the biosynthesis, transportation, and metabolism of polyamines. Of particular significance, RAS and BRAF mutations are prevalent in THCA, exerting a close association with the onset and progression of this malignancy [21, 22].

Currently, several articles have confirmed the significant involvement of polyamine metabolism in both tumor cells and tumor‐related immune cells [24, 25]. However, the exact mechanism remains unclear. This study aimed to investigate the correlation between polyamine metabolism alterations in THCA and macrophage function. By elucidating a potential regulatory mechanism governing the interaction between macrophages in THCA, this research presents a novel treatment approach for locally advanced THCA.

## 2 Methods

### 2.1 Polyamine‐Associated Gene Retrieval

The genes associated with polyamines were sourced from the “REACTOME_METABOLISM_OF_POLYAMINES.v7.5.1.gmt” file retrieved from the GSEA‐MSigDB repository (http://www.gsea‐msigdb.org/gsea/index.jsp).

### 2.2 Expression Data Acquisition

Analysis of polyamine‐associated gene expression within the THCA dataset was conducted by accessing multiple TCGA datasets from the GDC portal (https://portal.gdc.cancer.gov). Single‐cell RNA‐seq data were procured from GSE184362, with quality control procedures detailed in the subsequent “Single‐Cell Data Analysis” section.

### 2.3 Single‐Cell Data Analysis

Quality control criteria for cells were established as follows: cell counts exceeding 200, mitochondrial content below 10%, and erythrocyte content below 3%. Seurat R package identified cells from seven tumor samples and five normal samples, categorizing them into nine distinct cell types: T cells, B cells, NK cells, epithelial cells, macrophages, smooth muscle cells, endothelial cells, fibroblasts, and plasma cells. The “LogNormalize” function in Seurat was utilized for normalizing single‐cell expression data. Principal component analysis (PCA) and t‐distributed stochastic neighbor embedding (T‐SNE) were employed for dimensionality reduction. Cell types were assigned based on the GEO series authors' annotations.

### 2.4 CellChat Analysis

The R package CellChat was employed to identify intercellular communications between malignant cells and other cell types in both normal and tumor groups. The “netVisual‐bubble” function visualized differences in receptor‐ligand profiles between normal and tumor cells.

### 2.5 Single‐Cell Gene Set Enrichment Analysis

The AUCell method was applied to assess the polyamine‐associated scores of single‐cell gene sets. The “AddModuleScore” function calculated these scores. ToppGene (https://toppgene.cchmc.org/enrichment.jsp) was utilized for pathway enrichment analysis of each cell type's marker genes. Additionally, the scMetabolism R package predicted metabolic scores for cell types.

### 2.6 Pseudotime Analysis

The monocle R package was used to uncover developmental trajectories within polyamines‐related score groups, with ggplot2 illustrating these trends.

### 2.7 Non‐Negative Matrix Factorization (NMF) Analysis

NMF analysis was conducted on 58 polyamine‐associated regulators in macrophages to observe the impact of polyamine‐mediated regulator expression, following established methodologies.

### 2.8 Microenvironment Cell Abundance and Immune Infiltration Calculation

CIBERSORT and ESTIMATE algorithms calculated MAM and TME scores, assessing immune checkpoints, cell proportions, and processes. Polyamines‐related subgroup signature scores were determined to evaluate microenvironment‐related signature distribution differences.

### 2.9 Immunotherapy Response Prediction in THCA

TIDE scores, available at http://tide.dfci.harvard.edu/, were used to predict sensitivity to PD1 and CTAL4 treatments within the submap platform, which analyzes expression profile similarities. The Cancer Immunome Atlas (TCIA) provided immune pheno score (IPS) data for THCA patients, with IPS differences between polyamines‐related subgroups analyzed using boxplots.

## 3 Results

### 3.1 Identification of the Polyamines‐Related Score in THCA in the Single Cell Level

**FIGURE 1:**
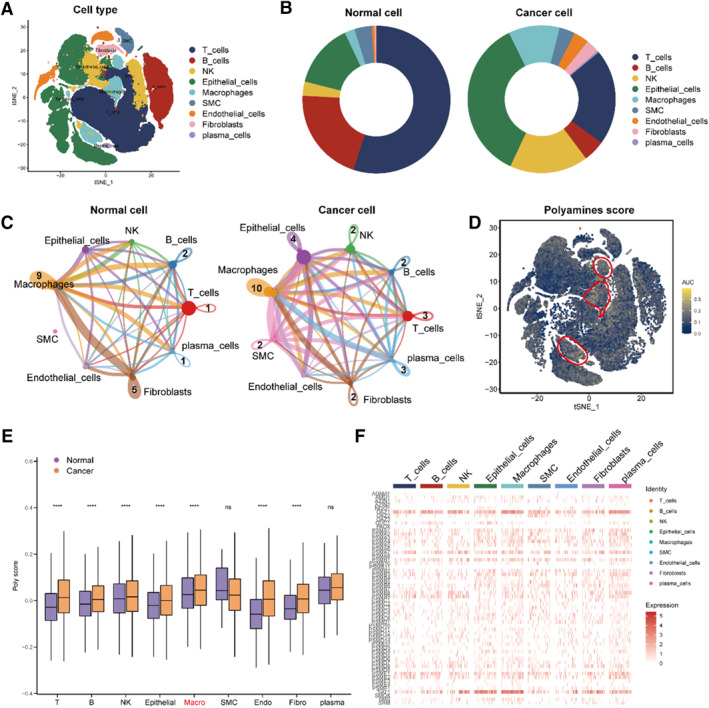
Identification of the Polyamines‐related score in THCA. (A) Cell type annotations by using the Seurat t‐distributed stochastic neighbor embedding (t‐SNE). (B) Cell types were compared from the tumor and normal cells by using the pie plot. (C) Cell–cell communications were compared from the tumor and normal cells. (D) Polyamines‐related score in the nine cell types by using the AUCell. (E, F) Polyamines‐related score in the nine cell types by using the AddModuleScore.

Firstly, the cells were divided into nine types: T cells, B cells, NK cells, epithelial cells, macrophages, smooth muscle cells, endothelial cells, fibroblasts and plasma cells, which was illustrated by t‐SNE function (Figure 1A). Then several cell types were compared from the tumor and normal cells by the pie plot and we found that several cell type ratios were elevated in tumor cells, such as epithelial cells, macrophages, and NK cells, while T and B cells were descended (Figure 1B). Additionally, we compared the cell‐chat analysis between the tumor and normal cell groups and found that macrophages play a vital role in the cell communication (Figure 1C). We analyzed polyamines‐related scores in 9 cell types at the single‐cell level by AUCell and found that polyamines‐related scores were highly expressed in macrophages (Figure 1D). In addition, the function “AddModuleScore” also proved that (Figure 1E,F). Hence, the process of polyamines may influence the macrophages of THCA.

### 3.2 Polyamines‐Mediated Macrophages Resembled Classical Features

**FIGURE 2:**
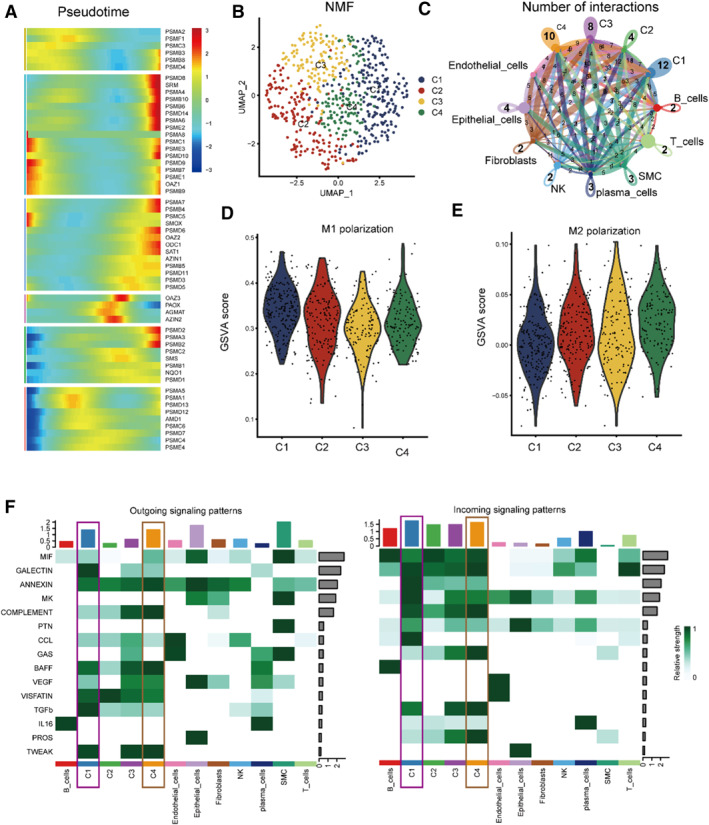
Polyamine—mediated macrophages resembled classical features. (A) Trajectory Analysis reveals the role of Polyamines in macrophages. (B) Macrophages are divided into four groups based on polyamines‐related gene expression by using NMF analysis. (C) The role in the cell‐communications of macrophages. (D, E) Evaluate the relationship between the macrophage features generated by polyamines and the existing macrophage features by using the Gene Set Variation Analysis (GSVA) function. (F) The signaling changes (the outgoing and incoming interaction) in various cell‐types.

In order to find the underlying developing potential of polyamines in macrophages, we used “monocle2” and found that amounts of polyamines‐related genes are important during the trajectory of macrophages (Figure 2A). Thus, NMF analysis was performed and the macrophages were divided into four groups on the basis of polyamines‐related gene expression (C1: PSME2+macro‐C1; C2: None‐macro‐C2; C3: None‐macro‐C3; C4: PSMA2‐macro‐C4, Figure 2B). Then, by the cell‐chat analysis, we found that the C1 and C4 groups are important in the cellular communication of macrophages (Figure 2C). Besides, to evaluate the relationship between the macrophage features generated by polyamines and the existing macrophage features, we assessed the degree of M1 and M2 polarization using the Gene Set Variation Analysis (GSVA) function; we found that group C1 showed a higher level of M1 polarization, while group C4 showed a higher level of M2 polarization (Figure 2D,E). Moreover, we examined changes in signals across a variety of cell types, and the results showed that some signals are critical in the strength of output and afferent interactions in C1 and C4 macrophages. For instance, the TGFB signaling pathway may occupy a central position in the outgoing interaction mode of C1‐macrophages, while the IL16 signaling pathway may occupy a central position in the afferent interaction mode of C4‐macrophages (Figure 2F).

### 3.3 Identification of the Polyamines‐Related Clusters and Their Potential Enriched Pathways at the Single‐Cell Level

**FIGURE 3:**
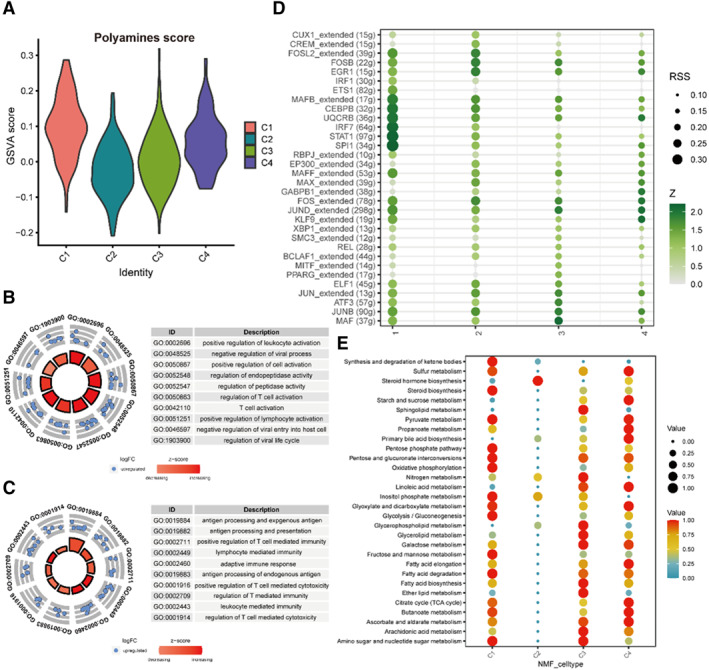
The relationship between the macrophage features generated by Identification of the Polyamines‐related clusters and their potential enriched pathways. (A) Polyamines‐related score in the C1‐C4 groups by using AddModuleScore. (B, C) Several immune pathways in the C1(B) and C4(C) groups by using GO analysis. (D) Specific TFs of C1‐C4. (E) The metabolism score for C1‐C4.

We first used the function “AddModuleScore” and found that the C1 and C4 groups acquired higher polymine scores (Figure 3A). Then GO analysis was utilized and we observed that the C1 and C4 groups both participated in several immune pathways, such as T cell activation, adaptive immune response, and regulation of T cell mediated cytotoxicity (Figure 3B,C). In addition, we independently calculated the metabolic scores of each cell, which also indicated that the C1 and C4 groups played an important role in the metabolic process (Figure 3E). In order to determine the potential specific TFs of each cell type, we demonstrated that SPI1 (34g), IRF7 (64g) and STAT1 (97g) may serve as regulators in the macrophages (Figure 3D).

### 3.4 Differences in the TME Infiltration Among Polyamines‐Related Subgroups

**FIGURE 4:**
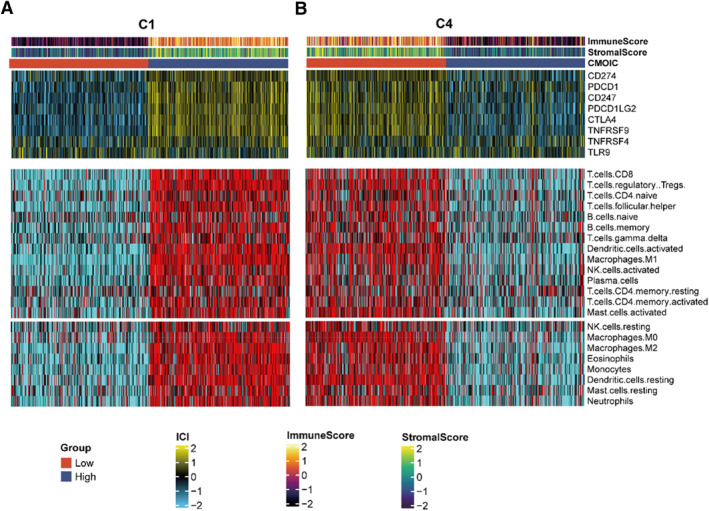
Differences in the TME infiltration among Polyamines‐related subgroups. (A) Immune scores, stromal scores, scores of the immune checkpoints, and immune cell types were determined to elucidate the tumor immune infiltration landscape between the low and high C1 subgroups. (B) Immune scores, stromal scores, scores of the immune checkpoints, and immune cell types were determined to elucidate the tumor immune infiltration landscape between the low and high C4 subgroups.

From the above results, we noticed that the C1 and C4 groups influenced the immune‐related pathways at the single cell level. To further analyze the changes of immune process in the C1 and C4 group, we downloaded the bulk‐RNA data from TCGA dataset. Then, Cibersort methods were used to determine immune scores, matrix scores, immune checkpoint scores and immune cell types to clarify the tumor immune invasion landscape between low, high C1 or C4 subgroups (Figure 4A,B).

### 3.5 Response to the Immune Checkpoint Block (ICB) Therapy Among Polyamines‐Related Subgroups

**FIGURE 5:**
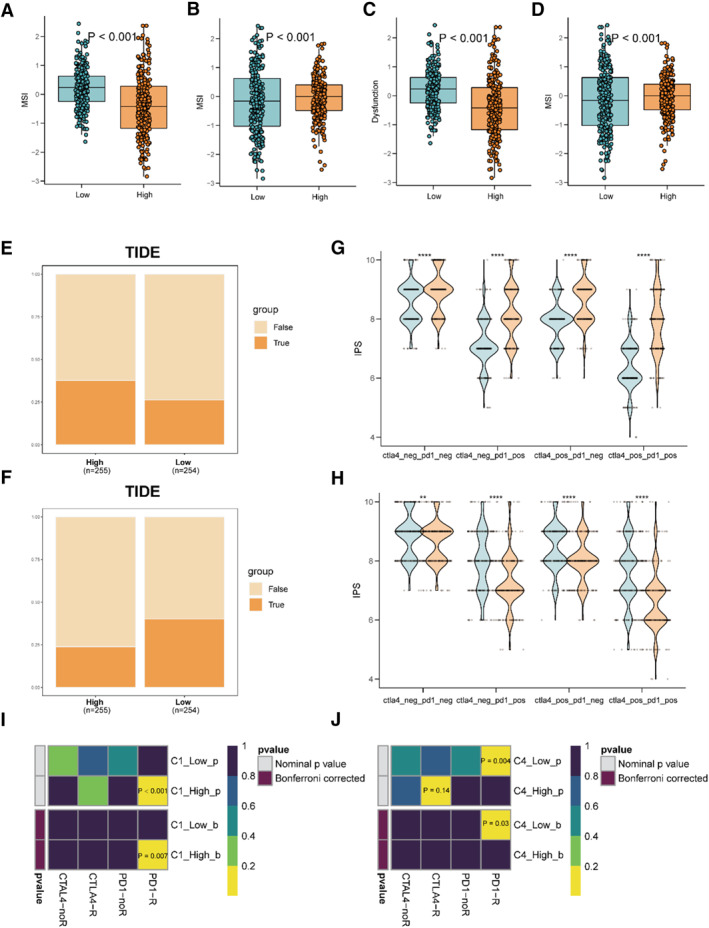
Response to the immune checkpoint block (ICB) therapy among Polyamines‐related subgroups. (A) The MSI among the low‐C1 subgroup and high‐C1 subgroup. (B) The MSI among the low‐C4 subgroup and high‐C4 subgroup. (C) The dysfunction among low‐C1 subgroup and high‐C1 subgroup. (D) The dysfunction among low‐C4 subgroup and high‐C4 subgroup. (E) TIDE were used to assess the response of the high‐C1 and low‐C1 the ICB of PD1. (F) TIDE were used to assess the response of the high‐C4 and low‐C4 to the ICB of PD1. (G) Immunophenotype score (IPS) was used to predict the response of low‐C1 and high‐C1 to the ICB of PD1. (H) Immunophenotype score (IPS) was used to predict the response of low‐C4 and high‐C4 to the ICB of PD1. (I, J) The IPS of CTLA and PD1 in the groups was determined.

To further investigate the ICB therapeutic response in Polyamine‐associated subgroups, we used the TIDE platform for predicting the response to immune checkpoint inhibitors, which is widely used in various studies. The results demonstrated that the MSI and dysfunction were higher in the high‐C4 score subgroup, while lower in the low‐C1 subgroup, which is identical to the former results (Figure 5A–D). In addition, TIDE and submap platform showed that the high C1 and low C4 subgroups responded better to the ICB of PD1 (Figure 5E–J). Lastly, the IPS of CTLA and PD1 were measured in each group, indicating higher sensitivity in the high C1 subgroups and the low C4 subgroups (Figure 5G,H).

### 3.6 Higher Expression of Polyamine Metabolism Related Proteins in THCA Compared to Adjacent Tissues

**FIGURE 6:**
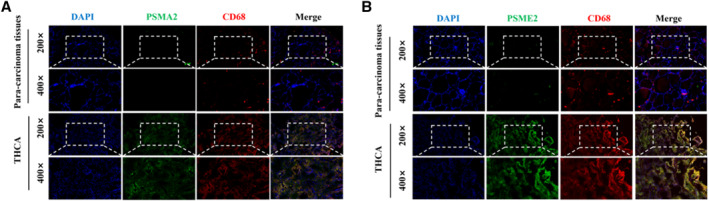
Higher expression of polyamine metabolism related proteins in THCA compared to adjacent tissues. (A) Immunofluorescence was used to verify changes in C4 group macrophages in cancer and adjacent tissues. (B) Immunofluorescence was used to verify changes in C1 group macrophages in cancer and adjacent tissues.

To verify the relationship between changes in polyamine metabolism in thyroid tissue and THCA tissue in clinical specimens, we used immunofluorescence to validate the expression of PSME2 and PSMA2 (Figure 6A,B). Our experiment showed that the content of PSME2 and PSMA2 in THCA tissue were apparent increased. To further validate the changes in macrophage polyamine metabolism, we added CD68 labeling of macrophages and found that the content of C1 and C4 subpopulations of macrophages in tumor tissue also significantly increased, which is consistent with our prediction. These data suggest that polyamine metabolism in macrophages is involved in progression, and polyamine metabolism in tumor cells may also play a major role in cancer progression.

### 3.7 Locally Advanced Patients Exhibit Higher Levels of Polyamine Metabolism

**FIGURE 7:**
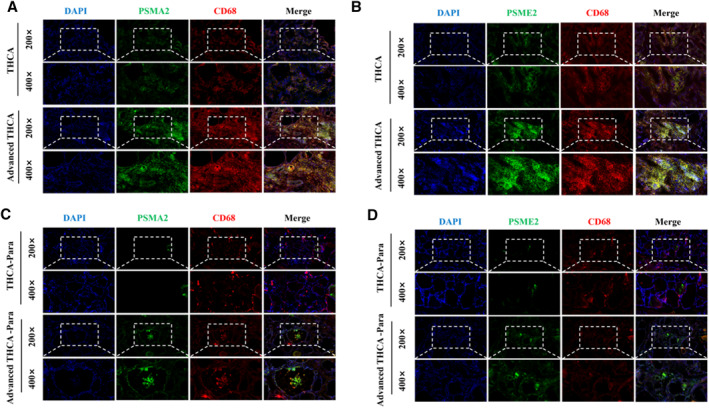
Locally advanced patients exhibit higher levels of polyamine metabolism. (A) Immunofluorescence was used to verify changes in C4 group macrophages in cancer and advanced cancer tissues. (B) Immunofluorescence is used to verify changes in C1 group macrophages in cancer and advanced cancer tissues. (C, D) Immunofluorescence is used to verify polyamine metabolism in adjacent tissues of early and advanced cancer.

To verify the specific role of macrophage and tumor cell polyamine metabolism in tumor progression, we detected the levels of PSME2 and PSMA2 in early THCA and locally invasive THCA tissue samples (Figure 7A,B). Interestingly, the levels of PSME2 and PSMA2 in advanced THCA are significantly higher than those in early stages, indicating that polyamine metabolism may be related to the invasion and proliferation of THCA. By increasing CD68 staining to confirm changes in macrophage subpopulations, it was found that the proportion of significantly increased C4 cells in advanced THCA was higher. Subsequently, we compared the expression of PSME2 and PSMA2 in tissues adjacent to advanced THCA and early THCA (Figure 7C,D). We found that although there was no significant difference in the expression of PSME2 and PSMA2 in macrophages, the content of PSME2 and PSMA2 in surrounding thyroid cells in advanced THCA was higher than that in tissues adjacent to early THCA. Therefore, polyamine metabolism may be involved in the composition of tumor microenvironment of THCA.

## 4 Discussion

Numerous studies have examined the important influence of macrophages on the occurrence and development of THCA [26, 27, 28]. However, research on the molecular mechanism underlying macrophage activity in THCA remains relatively scarce, impeding the development and application of targeted drugs for tumor‐related macrophages. Polyamine metabolism is critical for tumor survival and growth. Intracellular polyamines, such as putrescine and spermine, are essential cationic molecules that interact with negatively charged molecules. This interaction plays a pivotal role in mammalian signaling regulation, cell proliferation, differentiation, and immune system regulation. In tumor tissue, mutations in genes like KRAS, BRAF, and MYC impact polyamine metabolism, leading to an increase in polyamine content within the tissue. This elevation sustains tumor proliferation by upregulating polyamine biosynthesis and reducing catabolism [20, 21]. However, research specifically focused on polyamine metabolism in THCA tissue and its immune regulation in tumor‐related macrophages is currently limited, constraining the exploration of polyamine metabolism's role in the immune environment of THCA tumors. The present study addresses the active phenomenon of polyamine metabolism in THCA and further categorizes macrophages based on varying levels of polyamine metabolism. It investigates the correlation between polyamine metabolism and tumor macrophage‐related immunity within distinct subcellular populations. This approach offers promising avenues for advancing the treatment of locally advanced THCA.

Through data dimensionality reduction, we identified alterations in immune cell composition, particularly macrophages, in THCA tissue compared to normal tissue. Intriguingly, these changes in macrophage composition and immune function were found to be associated with heightened polyamine metabolism. We classified macrophages into four distinct groups, namely C1 to C4, based on variations in gene expression related to polyamine metabolism. Unlike traditional M1 and M2 classifications, our approach encompassed all macrophage types. Macrophages can differentiate into distinct functional phenotypes in response to microenvironmental signals, primarily the pro‐inflammatory M1 type and the anti‐inflammatory M2 type. Within tumor tissues, M2 macrophages contribute to tumor cell survival, while M1 macrophages play a significant role in tumor cell cytotoxicity. Notably, the subcellular population with high PSME2 expression (C1) exhibited similarities to M1 type macrophages, while the subcellular population with high PSMA2 expression (C4) displayed similarities to M2 type macrophages. On the other hand, C2 and C3 type macrophages showed lower activity in cellular communication within the tumor tissue compared to C1 and C4, yet they might still be involved in regulating tumor biological behavior. The specific regulatory mechanisms of C2 and C4 in tumors need to be further studied. Given the significance of immunotherapy for advanced THCA patients with poor response to iodine treatment, we delved into the relationship between changes in macrophage subpopulations and immunotherapy effectiveness. Our study grouped patients based on high or low C1 and C4 proportions to elucidate the distinct reactivity of macrophage subpopulations in tumor immunotherapy. The results revealed that the high C1/low C4 ratio group exhibited a significantly superior response to immunotherapy compared with the high C4/low C1 group. Interestingly, this treatment response correlated with the positivity of CTLA and PD1 loci. Notably, when both CTLA and PD1 loci were positive, the difference between the high C1/low C4 and high C4/low C1 groups became even more prominent. This finding offers a novel approach for clinically predicting immunotherapy efficacy and identifying suitable candidate populations for immunotherapy.

Polyamine metabolism holds promise not only in predicting immunotherapy efficacy and aiding patient population screening but also as a potential target for drug therapy in THCA. Notably, polyamines have demonstrated immunosuppressive properties, suggesting that targeted inhibition of polyamine metabolism in tumor tissue could enhance the anti‐tumor immune response. In a clinical setting, we compared PSME2 and PSMA2 expression levels between THCA patients and adjacent tissues, revealing significant differences in the two tissues. This indirectly highlights the importance of polyamine metabolism in sustaining tumor biological function. Moreover, through monitoring cancer tissue samples from both locally advanced and early‐stage THCA patients, we observed a certain correlation between excessive polyamine metabolism and tumor progression. Understanding the intricate relationship between polyamine metabolism and THCA pathogenesis provides valuable insights for potential therapeutic interventions and immunotherapy strategies.

Polyamine metabolism not only governs the immune phenotype of macrophages but also exerts regulatory effects on various other immune cell types [29, 30]. Numerous studies have paid close attention to the relationship between polyamine metabolism and the adaptive immune system, particularly exploring its impact on B cell activation and T cell activation in tumor tissue [31, 32]. B cells in tumor tissue can inhibit tumor development through various mechanisms, including initiating T cell‐mediated immune killing by producing tumor‐responsive antibodies. The development and activation of B cells are dependent on the expression of MYC and polyamine biosynthetic enzymes, with MYC being crucial for ODC1 expression [21, 33, 34]. Interestingly, research has shown that supplementing with spermine can reduce the apoptosis of activated B cells, highlighting the complex relationship between polyamines and B cell function in the context of tumor killing [35]. Polyamine metabolism's immune regulatory function in tumor tissue may primarily be achieved by influencing T cells, macrophages, and dendritic cells [36, 37, 38, 39]. Under the influence of polyamine metabolism, B lymphocytes become less susceptible to apoptosis and induce T cell receptor (TCR) activation in T cells [40, 41]. TCR activation triggers the conversion of arginine to ornithine and guanidine, promoting humic acid production [42]. Subsequently, the products of polyamine metabolism modulate T cell functional activity. Spermidine, for instance, fosters the transformation of T cells into immunomodulatory phenotypes through the FOXP3 pathway, reducing inflammatory killing effects [37, 43]. Additionally, polyamine metabolism impacts the epigenome and tricarboxylic acid cycle, thereby regulating the functions of helper T cells (TH1, TH2, TH17) and regulatory T cells (Treg) [36, 44]. Overall, given its role in TCR signaling and promotion of the Treg cell phenotype, polyamine metabolism is vital in tumor immunity. In the immunosuppressive microenvironment of tumors, the proportion of immunosuppressive dendritic cells and M2 macrophages tends to increase significantly, and these cell types heavily rely on polyamine metabolism to suppress the immune system. Consequently, targeting polyamine metabolism holds promise as an effective complementary therapy for immunotherapy of advanced tumors.

This study represents a preliminary investigation into the impact of polyamine metabolism on tumor‐related macrophages in THCA. However, it is important to acknowledge the limitation in our analysis, primarily due to the insufficient number of clinical samples. Consequently, the conclusions drawn from this study should be validated through the inclusion of a larger and more diverse set of clinical samples. Furthermore, to gain deeper insights into the mechanisms of polyamine metabolism and its regulation in macrophages, it is essential to establish an in situ tumor forming animal model. This model would allow for a more comprehensive exploration of the interactions and effects of polyamine metabolism on macrophage behavior within the context of THCA. Despite these limitations, our study has shed light on the distinctive characteristics and functional implications of polyamine metabolism in different macrophage subpopulations within THCA. This novel perspective opens up possibilities for targeted polyamine metabolism therapy in the treatment of advanced THCA. Nevertheless, further research and extensive clinical validation are necessary to fully comprehend the potential therapeutic implications of our findings.

## Author Contributions

Haoran Ding, Lingling Xue, and Shaoshi Zhu drafted the main manuscript. Haozhen Ren and Dayu Chen designed the research and prepared the figures. Ning Tang, Guang Zhang, Huan Gui, and Ziyu Wang contributed to the manuscript revision. All authors reviewed and approved the final manuscript.

## Ethics Statement

This research protocol was approved by the Human Research Ethics Committee of Nanjing Drum Tower Hospital Clinical College of Nanjing Medical University (No. 2023‐345‐01).

## Consent

All the patients gave written informed consent according to the Declaration of Helsinki, and all experimental methods were conducted following relevant guidelines.

## Conflicts of Interest

The authors declare no conflicts of interest.

## Data Availability

The datasets generated during the current study are available from the corresponding author on reasonable request.
